# Epigenetic silencing of *SMOC1* in traditional serrated adenoma and colorectal cancer

**DOI:** 10.18632/oncotarget.23523

**Published:** 2017-12-20

**Authors:** Hironori Aoki, Eiichiro Yamamoto, Akira Takasawa, Takeshi Niinuma, Hiro-O Yamano, Taku Harada, Hiro-O Matsushita, Kenjiro Yoshikawa, Ryo Takagi, Eiji Harada, Yoshihito Tanaka, Yuko Yoshida, Tomoyuki Aoyama, Makoto Eizuka, Akira Yorozu, Hiroshi Kitajima, Masahiro Kai, Norimasa Sawada, Tamotsu Sugai, Hiroshi Nakase, Hiromu Suzuki

**Affiliations:** ^1^ Department of Molecular Biology, Sapporo Medical University School of Medicine, Sapporo, Japan; ^2^ Department of Gastroenterology and Hepatology, Sapporo Medical University School of Medicine, Sapporo, Japan; ^3^ Department of Pathology, Sapporo Medical University School of Medicine, Sapporo, Japan; ^4^ Department of Digestive Disease Center, Akita Red Cross Hospital, Akita, Japan; ^5^ Department of Molecular Diagnostic Pathology, School of Medicine, Iwate Medical University, Morioka, Japan

**Keywords:** SMOC1, colorectal cancer, traditional serrated adenoma, DNA methylation, CIMP

## Abstract

Colorectal sessile serrated adenoma/polyps (SSA/Ps) are well-known precursors of colorectal cancer (CRC) characterized by *BRAF* mutation and microsatellite instability. By contrast, the molecular characteristics of traditional serrated adenoma (TSAs) are not fully understood. We analyzed genome-wide DNA methylation in TSAs having both protruding and flat components. We identified 11 genes, including *SMOC1*, methylation of which progressively increased during the development of TSAs. *SMOC1* was prevalently methylated in TSAs, but was rarely methylated in SSA/Ps (*p* < 0.001). RT-PCR and immunohistochemistry revealed that SMOC1 was expressed in normal colon and SSA/Ps, but its expression was decreased in TSAs. Ectopic expression of SMOC1 suppressed proliferation, colony formation and *in vivo* tumor formation by CRC cells. Analysis of colorectal lesions (*n* = 847) revealed that *SMOC1* is frequently methylated in TSAs, high-grade adenomas and CRCs. Among these, *SMOC1* methylation was strongly associated with *KRAS* mutation and CpG island methylator phenotype (CIMP)-low. These results demonstrate that epigenetic silencing of *SMOC1* is associated with TSA development but is rarely observed in SSA/Ps. SMOC1 expression could thus be a diagnostic marker of serrated lesions, and *SMOC1* methylation could play a role in neoplastic pathways in TSAs and conventional adenomas.

## INTRODUCTION

Colorectal cancer (CRC) is one of the most commonly diagnosed malignancies and a leading cause of cancer mortality. Appropriate screening and removal of premalignant lesions at high risk to develop CRC is essential for reducing the incidence and mortality of CRC. Identification of the molecular alterations that occur at the premalignant step would improve our understanding of the tumorigenesis mechanism and could lead to the development of new strategies for CRC prevention.

CRCs are thought to arise from adenomas through accumulation of multiple genetic alterations, including mutation of *APC*, *KRAS* and *TP53*. In addition to the adenoma-carcinoma sequence, the serrated neoplastic pathway is now recognized to be an alternative pathway of CRC development [1, 2]. Serrated lesions (serrated polyps) are largely subdivided into hyperplastic polyps (HPs), sessile serrated adenoma/polyps (SSA/Ps) and traditional serrated adenomas (TSAs), and the evidence now indicates that SSA/Ps and TSAs are important premalignant lesions. [3] It is well documented that SSA/Ps are precursors of sporadic CRCs with *BRAF* mutation, microsatellite instability (MSI) and concurrent hypermethylation of multiple loci, which is termed as CpG island methylator phenotype (CIMP) [2, 4–7]. TSAs are thought to be precursors of microsatellite stable (MSS) CRCs [8], but the molecular mechanisms underlying the development and progression of TSAs are not yet fully understood.

TSAs exhibit distinct genetic and epigenetic characteristics. For instance, recent studies showed that *PTPRK-RSPO3* fusion and *RNF43* mutations are characteristic features of TSAs [9, 10], although another study reported that *RNF43* mutations are observed in both SSA/Ps and TSAs [11]. SSA/Ps are tightly associated with *BRAF* mutation, while TSAs mostly harbor *BRAF* or *KRAS* mutations [8, 12]. TSAs with *BRAF* mutation exhibit features similar to SSA/Ps, including proximal colon location and CIMP-high. However, they rarely exhibit *MLH1* methylation, and they retain MLH1 protein expression [8]. The frequency of CIMP-high is lower in *KRAS* mutant and *KRAS*/*BRAF* wild-type TSAs, but approximately half of these lesions exhibit CIMP-low [8]. Another study also showed that TSAs are characterized by distal colon location, less frequent *BRAF* mutation and smaller numbers of methylated genes than SSA/Ps [13]. Taken together with recent observations that both *KRAS* and *BRAF* mutations can induce CIMP [14, 15], these results suggest that aberrant DNA methylation is an important driver of the serrated neoplastic pathway, though genes with aberrant methylation likely differ between SSA/Ps and TSAs.

Analysis of gene expression signatures in a large cohort of colon cancers suggested that this disease can be subcategorized into three molecular subtypes: colon cancer subtype 1 (CCS1) and CCS2 are associated with chromosomal instability and MSI, respectively, while CCS3 is characterized by an unfavorable prognosis and is thought to develop though the serrated neoplastic pathway [16]. It is uncertain whether CCS3 tumors originate from TSAs, but the lack of MSI and the relative abundance of *BRAF*/*KRAS* mutations and CIMP in this category suggests TSAs have the potential to develop aggressive CRCs. Thus, clarification of the underlying molecular alterations in TSAs may lead to development of useful biomarkers and new strategies for CRC prevention.

In earlier studies, we showed that analysis in lesions consisting of premalignant and more advanced components within the same tumors is a useful strategy for unraveling the molecular evolution during colorectal tumorigenesis [5, 7]. In the present study, we aimed to use that approach to clarify the molecular events that occur during TSA development. We identified a series of genes, including *SMOC1*, as targets of aberrant DNA methylation in TSAs and found that *SMOC1* methylation may be associated with the development and malignant progression of TSAs.

## RESULTS

### Methylation of *SMOC1* is acquired during the development of TSAs

**Figure 1:**
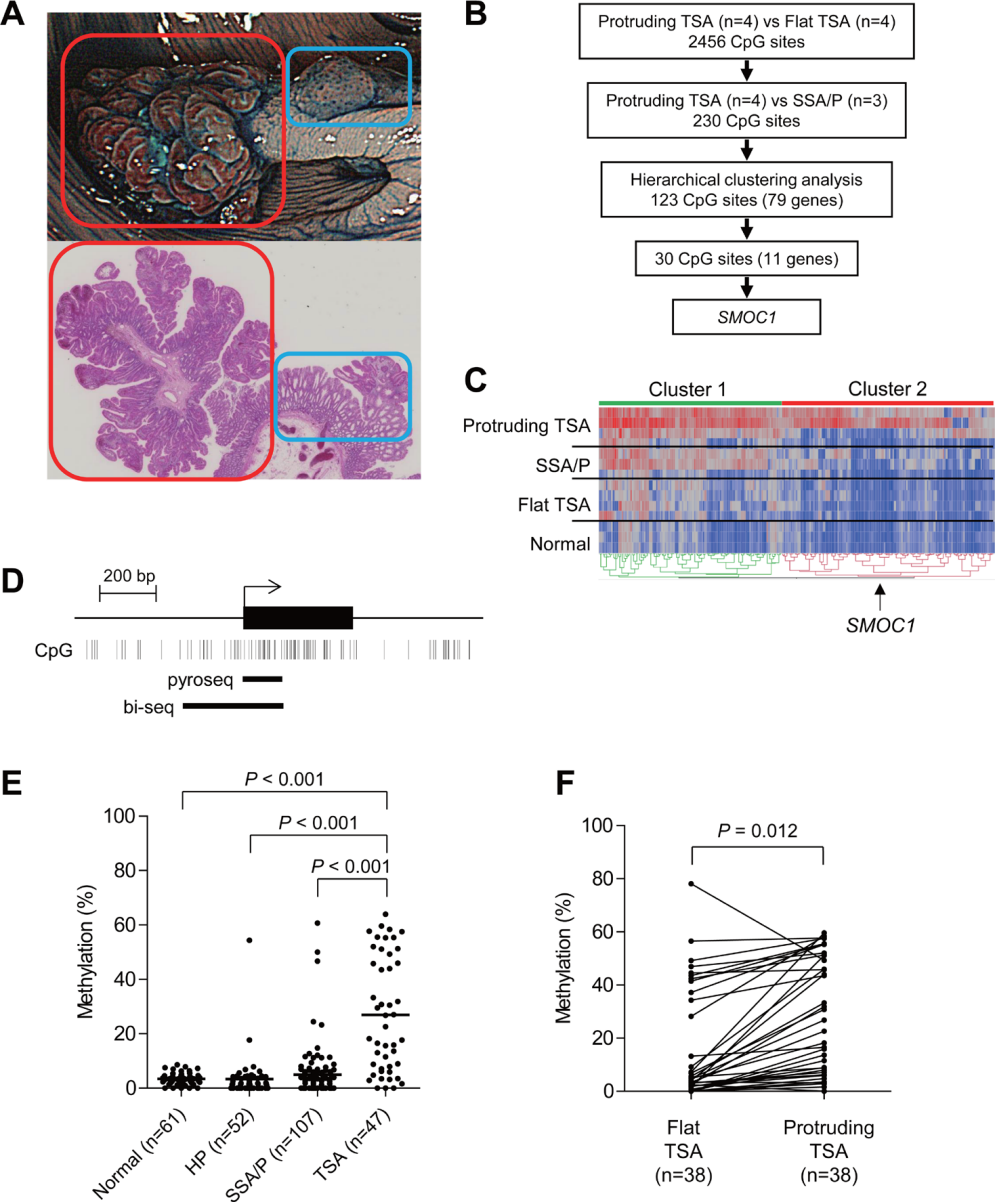
Identification of DNA methylation associated with the development of TSAs **(A)** Representative example of TSA. An endoscopic view is shown on the top and a histological view is below. Red boxes indicate a protruding component and blue boxes indicate a flat component. **(B)** Workflow to identify DNA methylation in TSAs. **(C)** Heatmap of the selected 230 CpG sites within the protruding components of TSAs (*n* = 4), SSA/Ps (*n* = 3), flat components of TSAs (*n* = 4) and normal colonic tissue (*n* = 3). Clusters 1 and 2 include 107 and 123 CpG sites, respectively. **(D)** Diagram of the promoter region of *SMOC1*. The transcription start site and exon 1 are shown on the top, and the regions analyzed using bisulfite pyrosequencing and bisulfite sequencing are shown below. **(E)** Summarized results of bisulfite pyrosequencing of *SMOC1* in specimens from the indicated lesions and adjacent normal colonic tissues. **(F)** Results of bisulfite pyrosequencing of *SMOC1* in specimens from TSAs consisting of protruding and flat components.

TSAs with protruding forms are often accompanied by flat components within the same tumors. The flat components are histologically similar to HPs and SSA/Ps, and are considered to be precursors of protruding TSAs (Figure 1A) [12, 17–19]. To identify changes in the DNA methylation that occur during the development of TSAs, we carried out Infinium HumanMethylation 450 BeadChip (HM450) analysis with a series of TSAs (*n* = 4) that contained both protruding and flat components. To identify DNA methylation specifically associated with TSA development, we also analyzed a set of SSA/P specimens (*n* = 3) using HM450. We first compared the methylation status between the protruding and flat components of the TSAs, and identified a series of 2486 CpG sites at which methylation levels were significantly higher in the protruding portions (*p* < 0.05, |Δβ-value| > 0.1, Figure 1B, Supplementary Figure 1A). We then compared the methylation status of the selected 2456 CpG sites between the protruding TSAs and SSA/Ps, and identified a set of 230 CpG sites that were predominantly methylated in TSAs (Figure 1B, Supplementary Figure 1B).

Unsupervised clustering analysis using the selected 230 CpG sites revealed that they could be subcategorized into 2 clusters (Figure 1C). In the first cluster, we noted that methylation levels were elevated throughout the entire series of selected CpG sites in the protruding TSAs, whereas elevated methylation in the second cluster was more specific to the protruding TSAs (Figure 1C). Among the CpGs in cluster 2, examination of those located within CpG islands in gene promoters revealed that 30 CpGs were associated with the promoters of 11 genes, *B3GALNT1*, *CADPS*, *CCDC180*, *FAM92A1*, *FEZ1*, *FRMD4B*, *GABRA4*, *OGFRL1*, *PRDM16*, *SMOC1* and *ZNF345*. We next used bisulfite pyrosequencing to analyze the methylation status of these genes in additional clinical specimens that included normal colonic tissue (*n* = 61), HPs (*n* = 52), SSA/Ps (*n* = 107) and TSAs (*n* = 47). We found that the CpG island in *SMOC1* (SPARC-related modular calcium binding 1) was specifically methylated in TSAs (Figure 1D, 1E). Methylation of the remaining 10 genes was also elevated in TSAs, but the methylation was not specific to TSAs or was much less frequent than in *SMOC1* (Supplementary Figure 2). Moreover, bisulfite sequencing analysis in TSAs with protruding and flat components within the same tumors again revealed increased *SMOC1* methylation in the protruding components (Figure 1F). These results suggest methylation of *SMOC1* is associated with the development of TSAs.

### Methylation of *SMOC1* is associated with gene silencing

**Figure 2:**
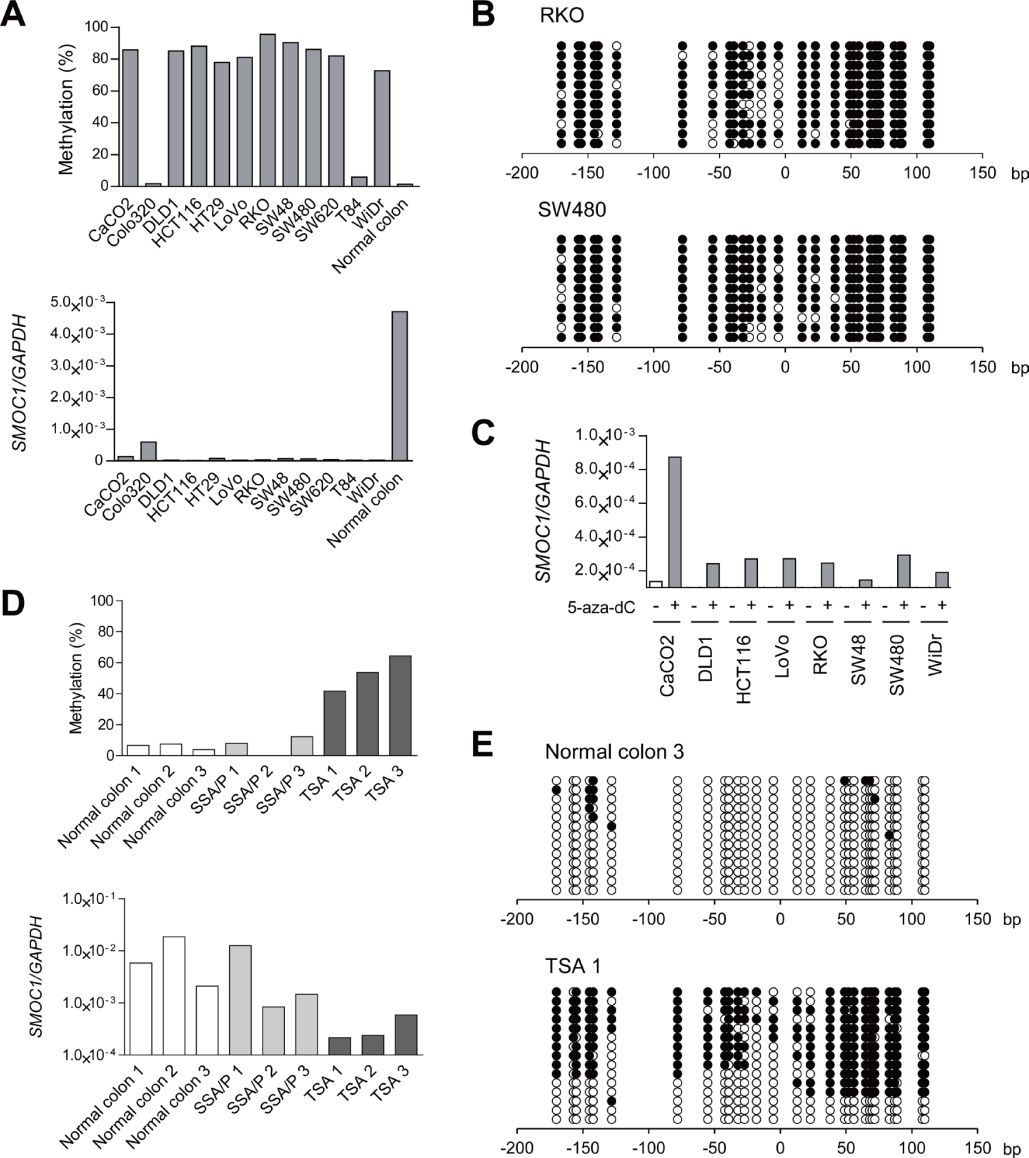
Analysis of *SMOC1* methylation and expression in colorectal tumors **(A)** Bisulfite pyrosequencing (top) and qRT-PCR (bottom) analyses of *SMOC1* in the indicated CRC cell lines and normal colonic tissue. **(B)** Bisulfite sequencing analysis of the *SMOC1* CpG island in the indicated CRC cell lines. Open and filled circles represent unmethylated and methylated CpG sites, respectively. **(C)** qRT-PCR analysis of *SMOC1* in the indicated CRC cell lines, with or without 5-aza-dC treatment. **(D)** Bisulfite pyrosequencing (top) and qRT-PCR (bottom) analyses of *SMOC1* in normal colonic tissues and primary serrated lesions. **(E)** Bisulfite sequencing analysis of *SMOC1* in the representative samples in (D).

To clarify whether *SMOC1* methylation is associated with its silencing, we analyzed the methylation and expression status of *SMOC1* in a series of CRC cell lines and samples of normal colonic tissue. Elevated *SMOC1* methylation was detected in 10 of the 12 CRC cell lines tested, whereas *SMOC1* was unmethylated in normal colonic tissue (Figure 2A, upper panel). Bisulfite sequencing analysis in selected CRC cell lines confirmed that the CpG island in *SMOC1* was densely hypermethylated in these cells (Figure 2B). Quantitative reverse-transcription PCR (qRT-PCR) analysis showed that levels of *SMOC1* expression were significantly decreased in all CRC cell lines tested, as compared to normal colonic tissue (Figure 2A, lower panel). Among the CRC cells, Colo320 and T84 cells exhibited only minimal levels of *SMOC1* methylation, and Colo320 showed the highest *SMOC1* expression among the CRC cell lines tested (Figure 2A). Moreover, *SMOC1* expression in multiple CRC cell lines was increased by the DNA methyltransferase inhibitor 5-aza-2′-deoxycytidine (5-aza-dC, 2 μM for 72 h) (Figure 2C). These results suggest *SMOC1* is epigenetically silenced in association with CpG island hypermethylation in nearly all of the CRC cell lines tested, though it may be inactivated by a different mechanism in T84 cells.

We next performed bisulfite pyrosequencing and qRT-PCR of *SMOC1* in clinical specimens that included normal colonic tissues, SSA/Ps and TSAs. Elevated methylation and decreased expression of *SMOC1* were detected in TSA specimens, whereas *SMOC1* was unmethylated and expressed in both normal colonic tissue and SSA/Ps (Figure 2D). Bisulfite sequencing revealed that the majority of CpG sites are unmethylated in normal colonic tissue (Figure 2E). By contrast, a TSA specimen showed a mixture of methylated and unmethylated alleles, probably reflecting a mixture of tumor and normal cells (Figure 2E). These results suggest *SMOC1* methylation is associated with its transcriptional silencing in TSAs.

**Figure 3:**
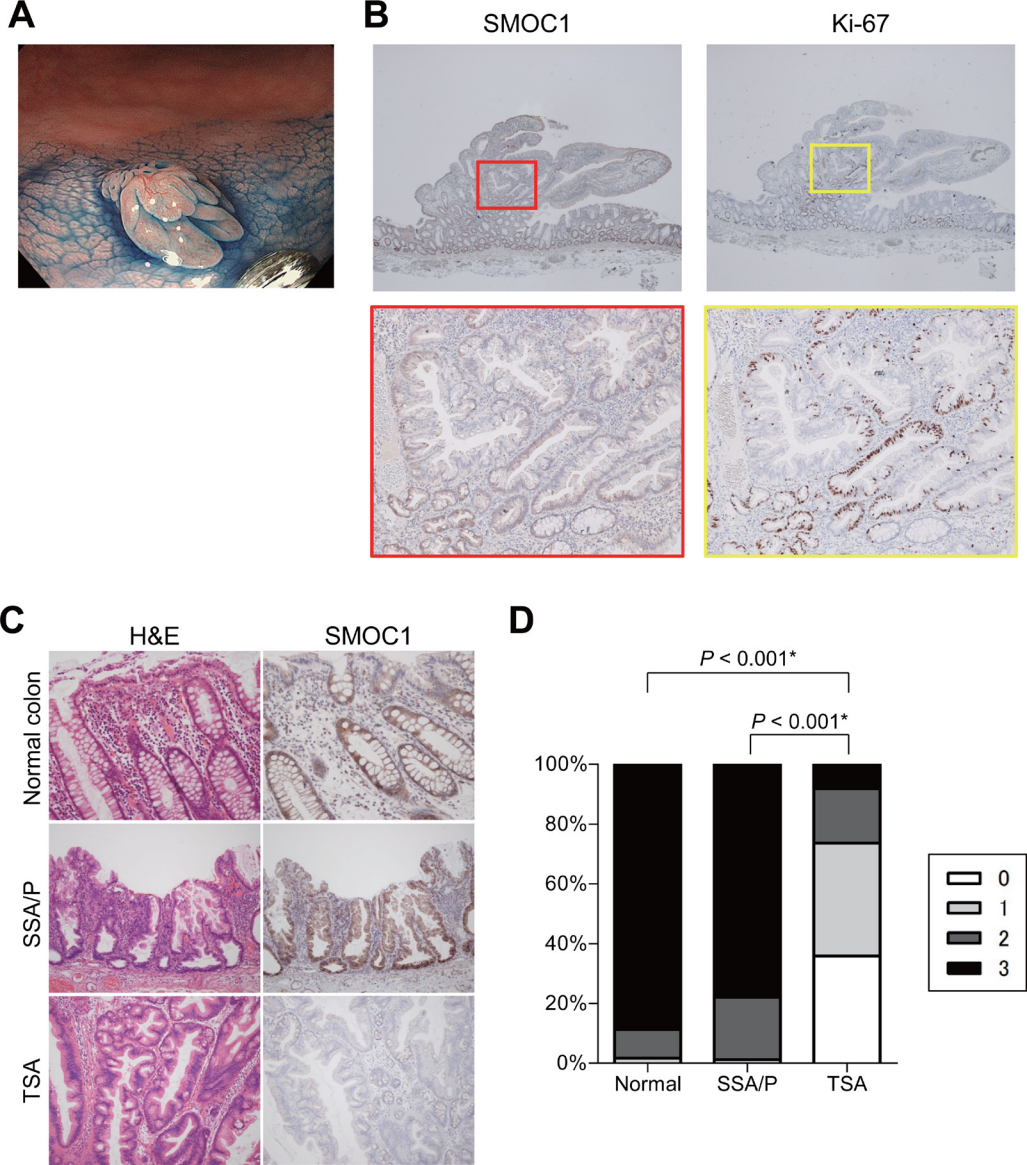
Immunohistochemical analysis of SMOC1 in serrated lesions **(A)** Endoscopic view of a TSA with protruding and flat components. **(B)** SMOC1 (left) and Ki-67 (right) staining in the TSA specimen shown in (A). Magnified views indicated by red and yellow boxes are shown below. **(C)** Representative views of hematoxylin and eosin (left) and SMOC1 (right) staining in normal colonic tissue, SSA/P and TSA specimens. **(D)** Summarized results for SMOC1 expression levels in normal colon (*n* = 23), SSA/Ps (*n* = 12) and TSAs (*n* = 11). ^*^Fisher's exact test.

We also evaluated SMOC1 expression in a TSA specimen with both flat and protruding components. Immunohistochemical analysis revealed SMOC1 expression to be lower in the protruding component than in the flat portion or adjacent normal mucosa (Figure 3A). Moreover, expression of Ki-67 was increased somewhat in the protruding component, suggesting loss of SMOC1 may be associated with increased cell proliferation (Figure 3B). In specimens of TSA (*n* = 11), SSA/P (*n* = 12) and adjacent normal colonic mucosa (*n* = 23), SMOC1 was expressed in the epithelium of normal colonic tissue and SSA/Ps, but SMOC1 expression was significantly reduced in a large number of TSAs (Figure 3C). Quantification using a scoring system revealed that there was little or no SMOC1 expression in the majority of TSA specimens tested (Figure 3D). These results indicate that SMOC1 may be a useful marker to discriminate between SSA/Ps and TSAs.

### Functional analysis of SMOC1 in CRC

**Figure 4:**
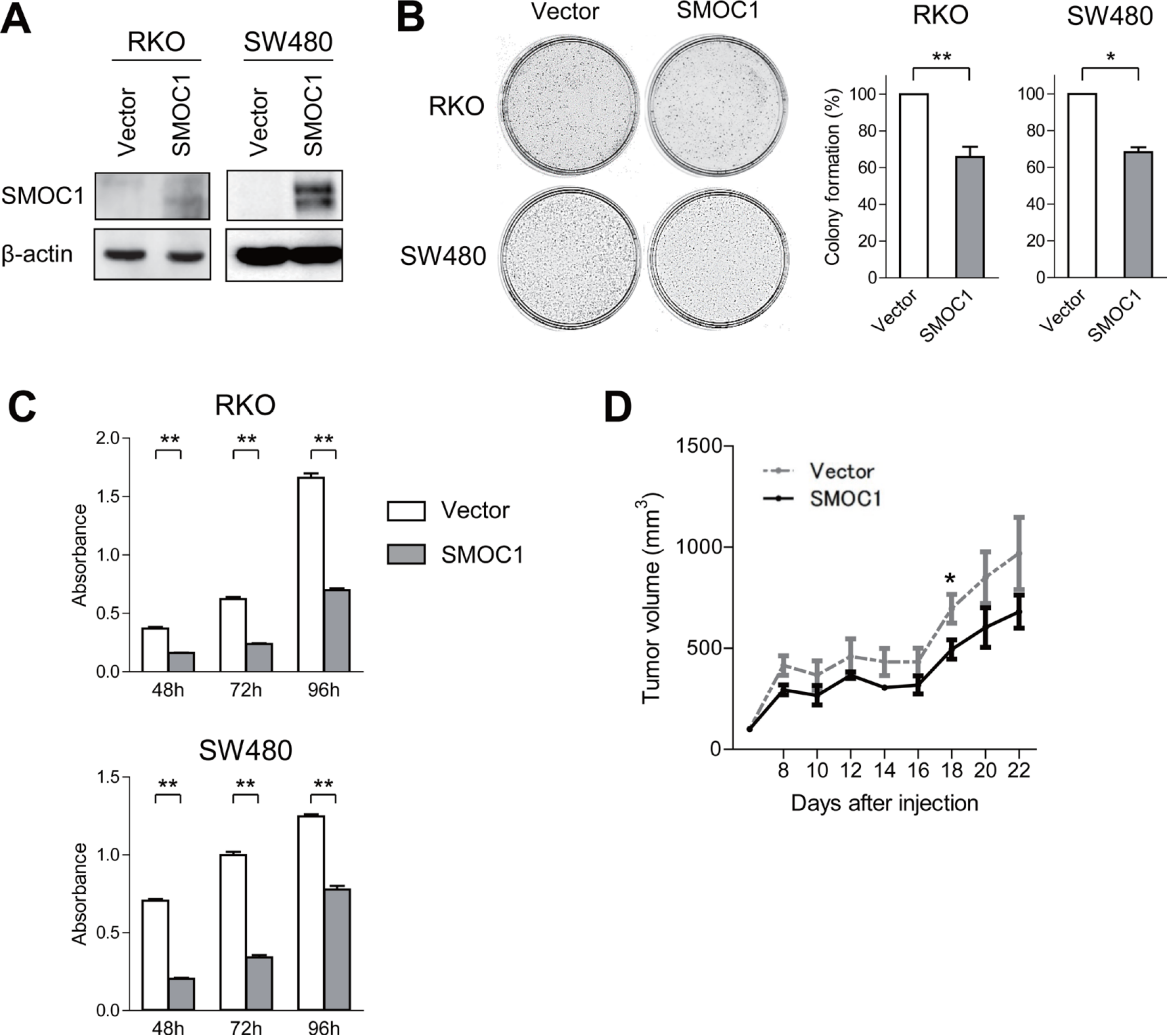
Functional analysis of SMOC1 in CRC cells **(A)** Western blot analysis of SMOC1 in the indicated CRC cells transfected with a SMOC1 expression vector or a control vector (Vector). **(B)** Colony formation assays using the indicated CRC cell lines transfected with the indicated vectors. Representative results are on the left, and relative colony formation efficiencies are on the right. Shown are means of 3 replications; error bars represent SDs. ^*^P < 0.05, ^**^P < 0.01. **(C)** Cell viability assays using the indicated CRC cell lines transfected with the indicated vectors. Shown are means of 8 replications; error bars represent SDs. ^**^P < 0.01. **(D)** Tumor growth in mice injected with SW480 cells transfected with the indicated vectors. Shown are means of 5 replications; error bars represent SDs. ^*^P < 0.05.

To investigate the function of SMOC1 in colorectal tumors, we transfected CRC cells with a SMOC1 expression vector or empty vector and confirmed the protein expression with western blotting (Figure 4A). We then carried out colony formation assays and found that SMOC1 suppresses colony formation by RKO and SW480 cells (Figure 4B), and in cell viability assays ectopic SMOC1 expression suppressed CRC cell proliferation (Figure 4C). On the other hand, SMOC1 did not suppress CRC cell migration or invasion in migration and Matrigel invasion assays (Supplementary Figure 3A, 3B). Flow cytometric analysis showed that SMOC1 also did not induce apoptosis in CRC cells (Supplementary Figure 3C). *In vivo*, inoculation of SW480 cells transiently transfected with a SMOC1 expression vector or empty vector into nude mice revealed that SMOC1 moderately suppresses tumor formation (Figure 4D, Supplementary Figure 3D).

Finally, we examined the effect of *SMOC1* depletion in cancer cells. Because all CRC cell lines only minimally expressed *SMOC1*, we analyzed *SMOC1* expression in a series of gastric cancer (GC) cell lines. qRT-PCR analysis revealed decreased *SMOC1* expression in the majority of GC cell lines, though not in MKN45 cells, which expressed *SMOC1* at a level similar to that in normal gastric tissue (Supplementary Figure 4A). However, *SMOC1* knockdown in MKN45 cells moderately promoted the cell proliferation (Supplementary Figure 4B, 4C). These results suggest *SMOC1* may act as a tumor suppressor.

### *SMOC1* methylation is associated with progression of colorectal tumors

To further clarify the role of *SMOC1* methylation in the development of CRC, we analyzed mixed colorectal lesions in which cancerous and TSA components were present together in the same tumors (Supplementary Figure 5A). The TSA components within these lesions were considered to be CRC precursors that might contain molecular alterations that promote malignant progression. We found that levels of *SMOC1* methylation were higher in TSAs associated with cancer than in those without cancer, suggesting *SMOC1* methylation may be a marker identifying TSAs at high risk of developing CRC (data not shown). We therefore generated a receiver operating characteristic (ROC) curve to assess the ability of *SMOC1* methylation levels to distinguish pure TSAs from TSAs with cancer and found the most discriminating cutoff value to be 36% (sensitivity, 75%; specificity, 66%) (Supplementary Figure 5B). By comparing the expression and methylation of *SMOC1* in representative clinical specimens, we confirmed that methylation levels higher than 36% were associated with decreased expression (Supplementary Figure 5C, 5D).

We also examined the association between *SMOC1* methylation and the clinicopathological features in a large cohort of patients with non-invasive colorectal tumors (*n* = 473). We then categorized the tumors according to their *SMOC1* methylation levels using the cutoff value calculated above, and found that elevated *SMOC1* methylation (≥36%) was associated with older age and larger tumor sizes, whereas it was not associated with gender, tumor location or tumor morphology (Table 1). We also found that *SMOC1* methylation was frequently elevated in colorectal adenomas and cancers. Notably, high *SMOC1* methylation was more prevalent in high-grade adenomas than low-grade adenomas (Table 1). The higher *SMOC1* methylation was associated with *KRAS* mutation, wild-type *BRAF*, *TP53* mutation and CIMP-low (Table 1). When we analyzed a series of invasive colorectal tumors (*n* = 374), we again found that elevated *SMOC1* methylation was associated with *KRAS* mutation and CIMP-low (Table 2). *SMOC1* methylation was also associated with older age and proximal colon location, but it was not associated with gender, tumor morphology, TMN category or lymphatic or vascular invasion (Table 2).

**Table 1:** Correlation between *SMOC1* methylation and the clinicopathological features of non-invasive colorectal tumors

*SMOC1* methylation			
	<36% (*n* = 376)	≥36% (*n* = 97)	*P*
Age (y, mean ± SD)	64.0 ± 11.1	68.6 ± 11.2	<0.001
Gender			
Female	112	31	NS
Male	264	66	
Location			
Proximal	186	50	NS
Distal	190	47	
Size (mm, mean ± SD)	10.6 ± 6.8	18.1 ± 13.0	<0.001
Morphology			
Depressed	2	1	NS
Flat	192	63	
Flat plus protruding	14	9	
Protruding	136	58	
Histology			
HP	51	1	<0.001
SSA/P	104	3	
SSA/P + CD	23	3	
SSA/P + cancer	11	3	
TSA	31	16	
TSA + cancer	0	8	
Low-grade adenoma	64	10	
High-grade adenoma	53	32	
Cancer	39	21	
*BRAF*			
Mut	151	19	<0.001
WT	225	78	
*KRAS*			
Mut	74	50	<0.001
WT	302	47	
*TP53*			
Mut	26	14	0.018
WT	350	83	
CIMP status			
Negative	248	57	0.010
Low	50	25	
High	78	15	
*MLH1* methylation			
Methylated	18	2	NS
Unmethylated	358	95	

NS, not significant; CD, cytological dysplasia; Mut, mutated; WT, wild type.

**Table 2:** Correlation between *SMOC1* methylation and the clinicopathological features of invasive colorectal tumors

*SMOC1* methylation			
	<36% (*n* = 235)	≥36% (*n* = 139)	*P*
Age (y, mean ± SD)	66.3 ± 12.2	70.6 ± 10.6	<0.001
Gender			
Female	84	64	NS
Male	151	75	
Location			
Proximal	86	71	0.006
Distal	149	68	
Morphology			
Type 0	44	18	NS
Depressed	30	8	
Flat	7	7	
Flat plus protruding	0	0	
Protruding	7	3	
Type 1	21	17	
Type 2	151	102	
Type 3	10	2	
Type 4	0	0	
Type 5	9	0	
*BRAF*			
Mut	19	4	NS
WT	216	135	
*KRAS*			
Mut	48	72	<0.001
WT	187	67	
*TP53*			
Mut	56	31	NS
WT	179	108	
CIMP status			
Negative	200	88	<0.001
Low	24	38	
High	11	13	
*MLH1* methylation			
Methylated	13	2	NS
Unmethylated	222	137	
pT category			
pT1	33	14	NS
pT2	32	28	
pT3	125	81	
pT4	25	9	
NA	20	7	
pN category			
pN0	140	83	NS
pN1	47	35	
pN2	22	13	
NA	26	8	
pM category			
pM0	184	120	NS
pM1	31	15	
NA	20	4	
Lymphatic invasion			
Negative	59	40	NS
Positive	140	85	
NA	36	14	
Vascular invasion			
Negative	85	58	NS
Positive	113	67	
NA	37	14	

NS, not significant; Mut, mutated; WT, wild type; NA, not available.

**Figure 5:**
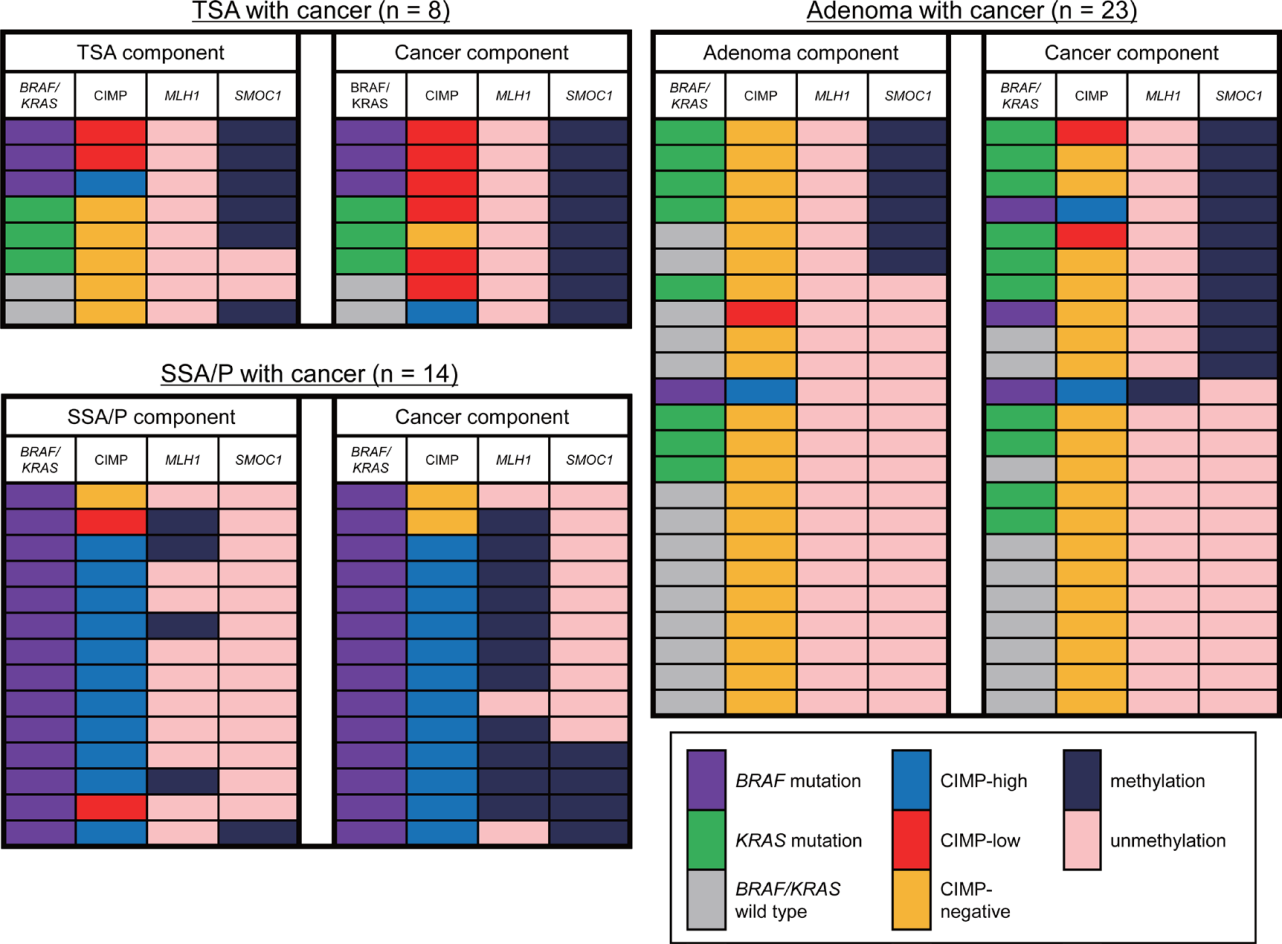
Changes in the molecular alterations during the progression of colorectal tumors Summary of the molecular features in colorectal tumors consisting of premalignant and malignant components are shown.

The results summarized above suggest that *SMOC1* methylation is associated with malignant progression of colorectal tumors, including TSAs and conventional adenomas. To test that possibility, we analyzed a series of colorectal lesions in which premalignant components were present together with cancerous components within the same tumors. As described above, *SMOC1* was methylated in most specimens of TSA with cancer (Figure 5). It is noteworthy that a majority of the cancer components exhibited CIMP-low, and none of the tumors showed *MLH1* methylation (Figure 5). Among the adenoma specimens with cancer (*n* = 23), elevated *SMOC1* methylation was found in 6 adenomas and 10 samples of cancer tissue (Figure 5). *SMOC1* methylation-positive adenomas and cancers were associated with frequent *KRAS* mutation and a lack of *MLH1* methylation. By contrast, the vast majority of SSA/Ps with cancer exhibited *BRAF* mutation and CIMP-high, while *SMOC1* methylation was infrequent in both the SSA/P and cancer tissues (Figure 5). The frequency of *MLH1* methylation progressively increased in the cancer tissues, which is consistent with our previous observations (Figure 5) [5, 7]. These results suggest that *SMOC1* methylation may play a key role in the development of TSAs and/or high-grade adenomas, which could eventually progress to CIMP-low or CIMP-negative CRCs.

## DISCUSSION

To clarify the molecular mechanism underlying the development of TSAs, we screened for DNA methylation changes associated with morphological and histological progression within TSA lesions. The list of genes we identified in this study included multiple genes implicated in cancer. For instance, *FEZ1* (also known as *LZTS1*) is a tumor suppressor gene located at 8p22, a region frequently deleted in human tumors [20]. *FEZ1* is silenced in association with promoter methylation in various malignancies, including gastric and breast cancer [21, 22]. A DNA methylome analysis in acute myeloid leukemia (AML) identified methylation of 7 genes, including *FAM92A1*, that were predictive of outcome in AML [23]. *PRDM16* (also known as *MEL1*) is overexpressed and associated with a poor prognosis in pediatric AML [24]. In addition, *PRDM16* is hypomethylated and overexpressed in astrocytoma [25], whereas it is hypermethylated in lung and esophageal cancers [26, 27].

Among the genes identified, we noted that methylation of *SMOC1* is highly specific to TSAs among serrated lesions. SMOC1 belongs to the SPARC (secreted protein acidic and rich in cysteine) family of matricellular proteins, which has 8 members: SPARC, SPARCL1/Hevin, SPOCK1, –2, –3, SMOC1, –2 and FSTL1 [28]. Although the physiological function of SMOC1 is not fully understood, recent studies have shown that SMOC1 is associated with osteoblast differentiation, ocular and limb development and angiogenesis [29–31]. Members of the SPARC family have been implicated in various human malignancies. SPARC, for instance, is markedly overexpressed in pancreatic cancer, and its expression is associated with a poorer prognosis [32, 33]. Conversely, *SPARC* is silenced by DNA hypermethylation in CRC, and its restoration using 5-aza-dC improves the response to chemotherapy [34]. Another study showed that exercise stimulates SPARC secretion from muscle tissues and that SPARC inhibits colon tumorigenesis [35]. These results suggest that SPARC may exert opposite effects during tumorigenesis in different tissues. In contrast to SPARC, FSTL1 was recently shown to inhibit pancreatic cancer growth [28]. *SMOC2* is known as an intestinal stem cell gene, and its elevation is associated with an aggressive and invasive phenotype in CRC [36]. In contrast to the other family members, few studies have shown possible involvement of SMOC1 in cancer. Brellier *et al.* reported that SMOC1 interacts with tenacin-C, an extracellular matrix protein overexpressed in various cancers, and that SMOC1 expression is elevated in brain tumors [37]. Fackler *et al.* searched for DNA methylation associated with hormone receptor status in breast cancer and found that methylation of 40 genes, including *SMOC1*, is associated with estrogen receptor status and disease progression [38]. However, the function of SMOC1 in breast cancer remains unknown, and *SMOC1* methylation has not been reported in other tumor types.

We showed here that *SMOC1* methylation is associated with transcriptional silencing in both primary and cultured colorectal tumor cells. Notably, *SMOC1* expression was significantly decreased in all CRC cell lines tested, and its ectopic expression suppressed proliferation of both CIMP-positive (RKO) and CIMP-negative (SW480) CRC cell lines. In addition to TSAs, *SMOC1* was frequently methylated in high-grade adenomas and CRCs, suggesting SMOC1 may play a common tumor suppressor role during colorectal tumorigenesis. However, the function of SMOC1 remains largely unknown, and further study will be necessary to clarify the biological significance of *SMOC1* methylation in colorectal tumors.

Histologically, TSAs often show a complex and distorted tubulovillous or villous configuration [39]. A recent study reported that serrated tubulovillous adenomas (sTVAs), which are a type of TVA, represent an important precursor of *KRAS* mutated, CIMP-low/negative and MSS CRCs [40]. In the present study, we noted that *SMOC1* methylation is strongly associated with *KRAS* mutation and CIMP-low, while it is not positively associated with *BRAF* mutation or *MLH1* methylation. These results indicate that *SMOC1* methylation may be associated with the development of *KRAS* mutant, CIMP-low and MSS CRCs derived from TSAs and sTVAs.

Our findings also demonstrated that immuno-histochemical staining of SMOC1 is highly discriminative between TSAs and SSA/Ps, suggesting SMOC1 could serve as a diagnostic marker for TSAs. Pathological diagnosis of serrated lesions is often confused by the morphological similarities between categories and the heterogeneity within respective lesions [17, 18]. A recent gene expression analysis in SSA/Ps and HPs identified ANXA10 as a potential marker of SSA/Ps [41]. The combination of SMOC1 and ANXA10 may provide improved diagnostic performance with serrated lesions, and further study is warranted.

In summary, we found that *SMOC1* is preferentially methylated in TSAs. Epigenetic silencing of *SMOC1* may be associated with the development of *KRAS* mutant, CIMP-low and MSS CRCs derived from TSAs or conventional adenomas. Our data also suggest that SMOC1 expression could serve as a diagnostic biomarker for serrated lesions, and that *SMOC1* methylation may be a predictive marker of precursor lesions at high risk of developing aggressive CRCs.

## MATERIALS AND METHODS

### Study population and cell lines

Colorectal tissue specimens were collected from Japanese patients who underwent endoscopic or surgical resection at Akita Red Cross Hospital and Teine-Keijinkai Hospital. Biopsy specimens were endoscopically obtained from a total of 991 specimens from 473 non-invasive tumors, 374 invasive tumors and 61 samples of adjacent normal colonic tissue. We carefully observed the microsurface structures of tumors using magnifying endoscopes and obtained biopsy specimens from tumor surfaces, so that the differences in the tumor cell content among samples would be minimal. Informed consent was obtained from all patients before collection of the specimens. Approval of this study was obtained from Institutional Review Board of Akita Red Cross Hospital, Teine-Keijinkai Hospital and Sapporo Medical University. CRC cell lines (CaCO2, Colo320, DLD1, HCT116, HT29, LoVo, RKO, SW48, SW480, SW620, T84 and WiDr) and a gastric cancer cell line (MKN45) were obtained and cultured as described previously [42, 43]. To restore epigenetically silenced genes, cells were treated with 2 μM 5-aza-2′-deoxycytidine (5-aza-dC) (Sigma-Aldrich, St Louis, MO, USA) for 72 h, replacing the drug and medium every 24 h. Genomic DNA was extracted using the standard phenol-chloroform procedure. Total RNA was extracted using TRIZOL reagent (Thermo Fisher Scientific, Waltham, MA, USA) and then treated with a DNA-free kit (Thermo Fisher Scientific).

### Endoscopic and histological analysis

Colorectal tumors were observed at high magni-fication using high-resolution magnifying endoscopes (CF-H260AZI; Olympus, Tokyo, Japan) after staining with indigo carmine dye and 0.05% crystal violet. Microsurface structures were classified according to Kudo's pit pattern classification system [44, 45]. Most often, one biopsy specimen was collected from each lesion for extracting genomic DNA. When more than two subcomponents were found in a single lesion, biopsy specimens were obtained for each portion, as described previously [5, 7]. The lesions were then treated through endoscopic mucosal resection, endoscopic submucosal dissection or surgical resection, after which histological analyses were carried out. Conventional adenomas were subcategorized into low-grade or high-grade adenomas. High-grade adenomas were defined as being 1 cm or more in diameter and/or with villous components and/or with high-grade dysplasia.

### Infinium assay

Genome wide DNA methylation was analyzed using an Infinium HumanMethylation450 BeadChip (Illumina, San Diego, CA, USA) as described [46]. The data were then assembled using GenomeStudio methylation software (Illumina) and analyzed using Microsoft Excel (Microsoft Corporation, Redmond, WA, USA) and JMP 11 (SAS Institute Inc., Cary, NC, USA). Probes on the X and Y chromosomes were excluded from the analysis. The Gene Expression Omnibus accession number for the microarray data is GSE96540.

### Methylation analysis using bisulfite pyrosequencing and bisulfite sequencing

Genomic DNA (1 μg) was modified with sodium bisulfite using an EpiTect Bisulfite Kit (Qiagen, Hilden, Germany), after which bisulfite sequencing and pyrosequencing were carried out as described previously [7]. Using five methylation markers (*MINT1*, *MINT2*, *MINT12*, *MINT31* and *p16*), tumors were defined as CIMP-high (four or more loci showed methylation), CIMP-low (two or three loci showed methylation) or CIMP-negative (0 or one loci showed methylation). For bisulfite sequencing, amplified PCR products were cloned into pCR2.1-TOPO vector (Thermo Fisher Scientific), and 10 to 15 clones from each sample were sequenced using an ABI3130x automated sequencer (Thermo Fisher Scientific). Primer sequences and PCR product sizes are listed in Supplementary Table 1.

### Mutation analysis

Mutations within codon 600 of *BRAF* and codons 12 and 13 of *KRAS* were examined by pyrosequencing using *BRAF* and *KRAS* pyro kits (Qiagen) according to the manufacturer's instructions. *TP53* mutation was initially assessed using PCR-SSCP followed by direct sequencing, as described previously [7].

### Quantitative reverse-transcription PCR

Single-stranded cDNA was prepared using SuperScript III reverse transcriptase (Thermo Fisher Scientific). Quantitative reverse-transcription PCR (qRT-PCR) was carried out using TaqMan Gene Expression Assays (*SMOC1*, Hs00951041_m1; *GAPDH*, Hs02758991_g1; Thermo Fisher Scientific) and a 7500 Fast Real-Time PCR System (Thermo Fisher Scientific). SDS ver. 1.4 (Thermo Fisher Scientific) was used for comparative delta Ct analysis.

### Immunohistochemistry

Immunohistochemical staining was performed as described previously [47]. A rabbit anti-SMOC1 polyclonal antibody (1:1000 dilution, C-20; Sigma-Aldrich) and a mouse anti-Ki-67 monoclonal antibody (1:100 dilution, Clone MIB-1; BioGenex, Fremont, CA, USA) were used. The staining intensity of SMOC1 was assessed as strong (3), moderate (2), weak (1) or negative (0). The proportions of positively stained tumor cells were recorded as 0 (no staining), 1 (1–10%), 2 (11–50%), 3 (51–80%) or 4 (81–100%). Because neoplasm heterogeneity caused varying degrees of immunoreactivity in the slides, we used an immunoreactive score (IRS) (e.g., intensity 3 × proportion 4 = immunoreactive score 12, scale of 0 to 12) to improve accuracy [47–49]. All slides were independently evaluated by two pathologists (AT, TA) who were blinded to the clinical data.

### Expression vector and siRNA for *SMOC1*

A full-length *SMOC1* cDNA was amplified by PCR using cDNA derived from *SMOC1*-expressing MKN45 cells as a template and then cloned into pcDNA3.2/V5/GW/D-TOPO (Thermo Fisher Scientific). Primer sequences are listed in Supplementary Table 1. For cell viability, migration, invasion, flow cytometry and xenograft assays, 1 × 10^6^ cells were transfected with 1 μg of control vector or SMOC1 expression vector using a Cell Line Nucleofector kit V (Lonza) with a Nucleofector I electroporation device (Lonza) as described [50]. For colony formation assays, 1 × 10^6^ cells in 6-well plates were transfected with 2.5 μg of the vectors using Lipofectamine 3000 (Thermo Fisher Scientific). For RNA interference (RNAi)-mediated *SMOC1* knockdown, 1 × 10^6^ cells in 6-well plates were transfected with 25 pmol of a Silencer Select Pre-designed siRNA targeting *SMOC1* (Thermo Fisher Scientific) or a negative control (Thermo Fisher Scientific) using Lipofectamine RNAiMAX (Thermo Fisher Scientific).

### Western blot analysis

Western blot analysis was performed as described previously [50]. A mouse anti-V5 monoclonal antibody (1:5000 dilution, R960-25; Thermo Fisher Scientific) and a mouse anti-β-actin monoclonal antibody (1:10000 dilution, clone AC-15; Sigma-Aldrich) were used.

### Colony formation assay

Cells were transfected with plasmids as described above. After incubation for 24 h, the transfectants were plated on 60-mm culture dishes and selected for 10 to 14 days in 1.0 mg/ml G418, after which colonies were stained with Giemsa.

### Cell viability assay

Cells were transfected with plasmids or siRNAs as described above. The transfectants were seeded into 96-well plates to a density of 5 × 10^3^ cells per well and incubated for 72 h. Cell viability assays were then carried out using a Cell Counting kit-8 (Dojindo, Kumamoto, Japan) according to the manufacturer's instructions.

### Cell migration and invasion assay

Cells were transfected with plasmids as described above. Transwell chambers were used for cell migration (BioCoat Control Insert 24-well plate 8.0 μm; Corning Inc., Corning, NY, USA) and invasion analyses (BioCoat Matrigel Invasion Chamber 24-well plate 8.0 μm; Corning Inc.). Cells were harvested 24 h after transfection and resuspended in culture medium containing 1 mg/ml bovine serum albumin, after which 1 × 10^5^ cells were added to the upper chamber. Culture medium with 10% fetal bovine serum was added to the lower well. After incubation for 24 h at 37°C, migrating or invading cells on the lower surface of the filter were fixed and stained using a Diff-Quik staining kit (Sysmex, Tokyo, Japan). Cell numbers were determined microscopically by counting in five randomly selected fields per membrane.

### Flow cytometry

Cells were transfected with the plasmids as described above. After incubation for 72 h, apoptosis was measured using an ApoScreen Annexin V Apoptosis Kit (Southern Biotech, Birmingham, AL, USA). Briefly, 1 × 10^6^ cells were washed twice in cold PBS and then resuspended in cold binding buffer, after which 10 μL of Annexin V-FITC was added to 100 μL of the cell suspension. The mixture was then incubated for 15 min on ice in the dark before addition of 380 μL of cold binding buffer and 10 μL of propidium iodide. For each sample, data were acquired from a minimum of 1 × 10^5^ cells using a BD FACSCant II (BD Biosciences, Franklin Lakes, NJ, USA) with BD FACSDiva software (BD Biosciences), and were analyzed using FlowJo ver. 10 (FlowJo, LLC, Ashland, OR, USA).

### Xenograft studies

Experiments were performed in accordance with the protocols approved by the institutional animal ethical committee at Sapporo Medical University. Mixtures of 1 × 10^6^ SW480 cells transfected with control vector or SMOC1 expression vector and 0.2 ml of Matrigel basement matrix (Corning Inc. Corning, NY, USA) were injected subcutaneously in the areas of the bilateral thighs of 6-week-old BALB/cAJcl-nu mice. Tumor size was measured every 2 days using digital calipers, and tumor volume was calculated using the formula length × width^2^/2. The experiment was repeated twice.

### Statistical analysis

To compare differences in continuous variables between groups, *t* tests or ANOVA with post hoc Tukey's tests were performed. Fisher's exact test or chi-squared test was used for analysis of categorical data. Values of *P* < 0.05 (two-sided) were considered statistically significant. The minimum *P*-value method was used to determine the best cutoff value for the *SMOC1* methylation level. Statistical analyses were carried out using GraphPad Prism ver. 5.0.2 (GraphPad Software, La Jolla, CA, USA).

## SUPPLEMENTARY MATERIALS FIGURES AND TABLE




