# Supplementary material for: Epigenetic silencing of SMOC1 in traditional serrated adenoma and colorectal cancer
Source: Oncotarget. 2017 Dec 20;9(4):4707–21. doi: 10.18632/oncotarget.23523 (PMC5797007; doi:10.18632/oncotarget.23523)
Supplement: Supplementary file 1 [file oncotarget-09-4707-s001.pdf]

Epigenetic silencing of *SMOC1* in traditional serrated adenoma and colorectal cancer

SUPPLEMENTARY MATERIALS

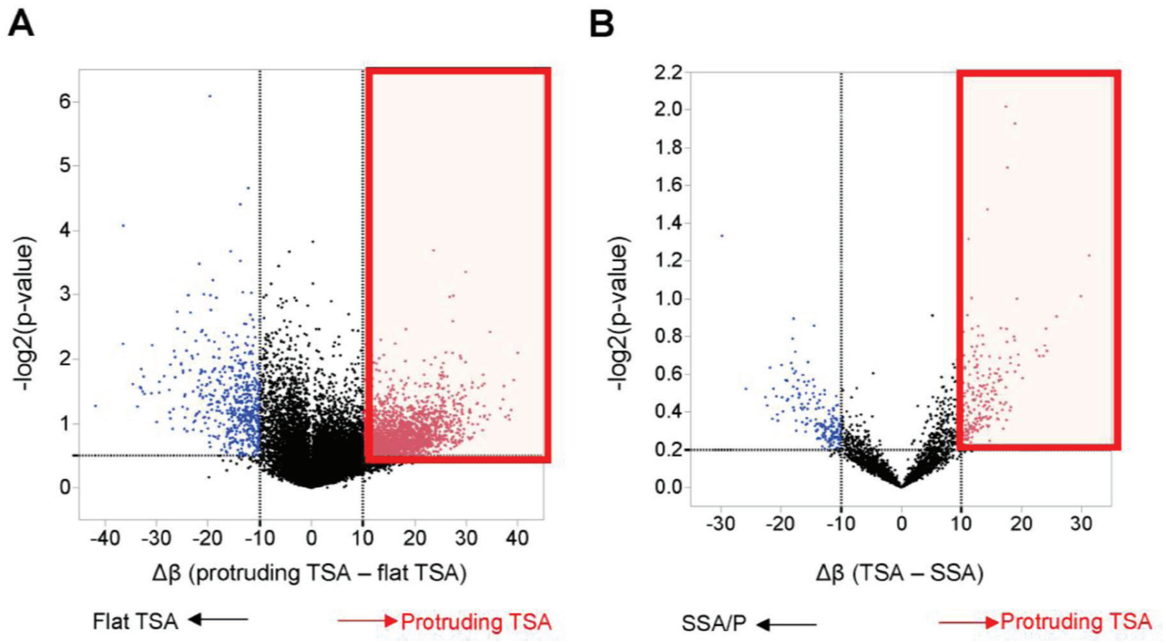

Supplementary Figure 1: Identification of DNA methylation associated with the development of TSAs. (**A**) Volcano plot showing the Infinium HumanMethylation 450 BeadChip data obtained from the protruding and flat components of TSAs. A set of 2456 CpG sites indicated by a red box were selected for further analysis. (**B**) Volcano plot showing the BeadChip data of the selected 2456 CpG sites obtained from protruding TSAs and SSA/Ps. A set of 230 CpG sites indicated by a red box were selected for further analysis.

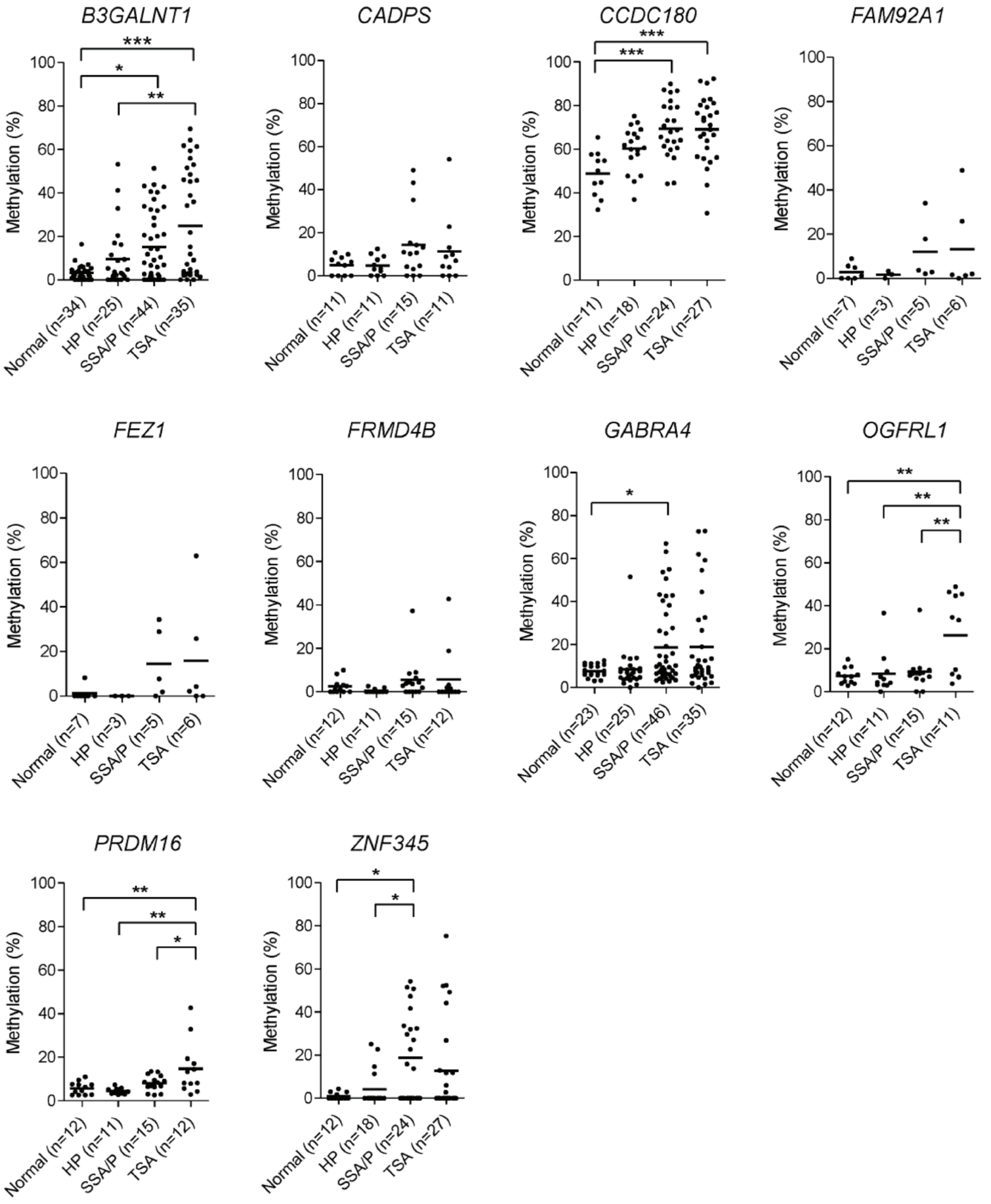

Supplementary Figure 2: Summary of the DNA methylation analysis of 10 selected genes. Results of bisulfite pyrosequencing analysis in indicated samples are shown. $^{*}P < 0.05$, $^{**}P < 0.01$, $^{***}P < 0.001$, Tukey-Kramer method.

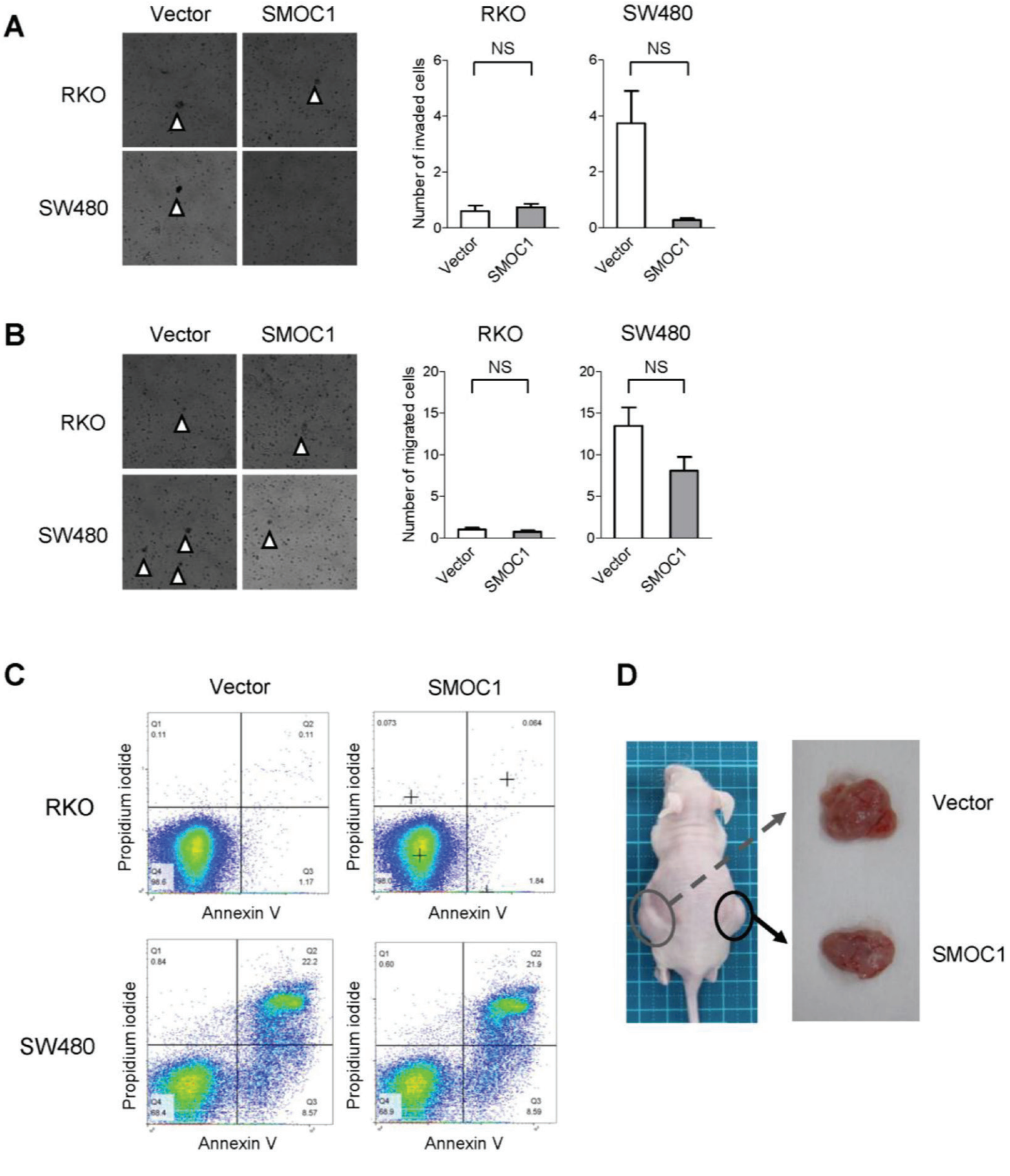

Supplementary Figure 3: Functional analysis of SMOC1 in CRC cells. (**A**) Matrigel invasion assays using the indicated CRC cell lines transfected with a SMOC1 expression vector or a control vector. Invading cells are indicated by arrowheads. Shown on the right are the means of 5 random microscopic fields per membrane; error bars represent SDs. NS, not significant. (**B**) Cell migration assays using the indicated CRC cell lines transfected with the indicated vectors. Migrating cells are indicated by arrowheads. Shown on the right are the means of 5 random microscopic fields per membrane; error bars represent SDs. (**C**) Representative results of apoptosis analysis in CRC cells transfected with a SMOC1 expression vector or a control vector. (**D**) Representative tumor xenografts in a nude mouse. SW480 cells transfected with the indicated vectors were injected subcutaneously into the thigh areas of nude mice. After 22 days, the mice were sacrificed and xenografts were excised.

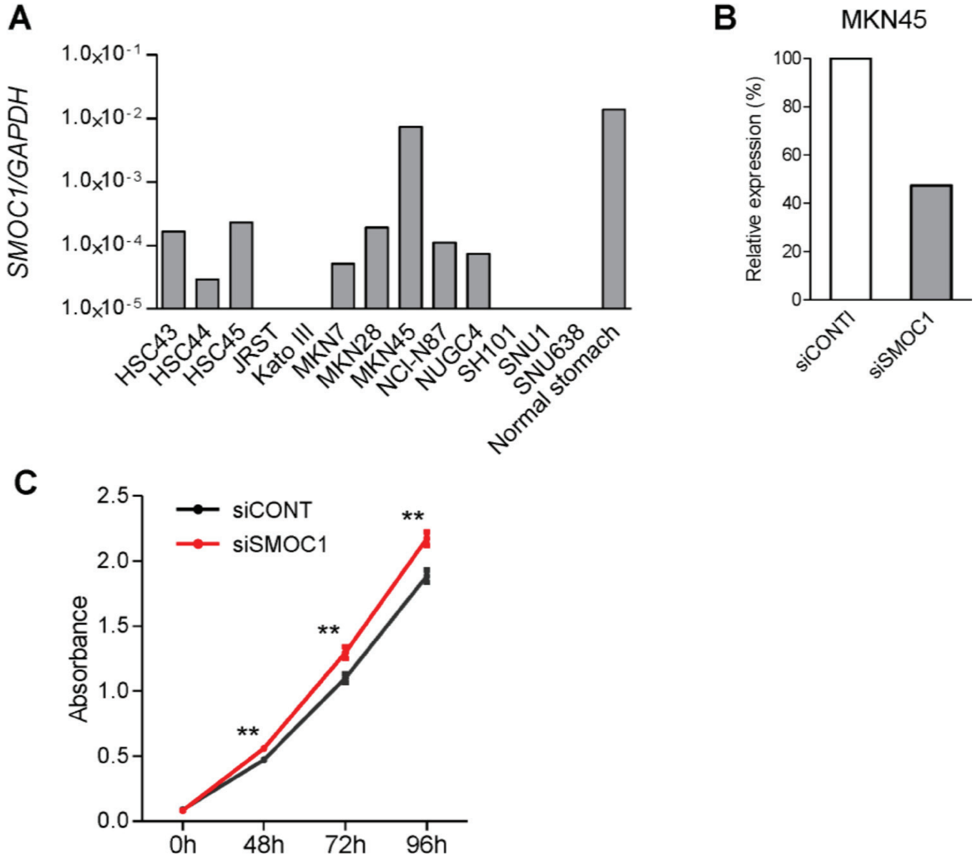

Supplementary Figure 4: *SMOC1* knockdown promotes gastric cancer cell proliferation. (**A**) RT-qPCR analysis of *SMOC1* in the indicated gastric cancer cell lines and normal stomach tissue. (**B**) RT-qPCR of *SMOC1* in MKN45 cells transfected with control siRNA (siCONT) or a siRNA targeting *SMOC1* (siSMOC1). Values were normalized to cells transfected with the control siRNA. (**C**) Cell viability assays using MKN45 cells transfected with the indicated siRNAs. Results are means of 8 replications; error bars represent SDs. $^{**}P < 0.01$.

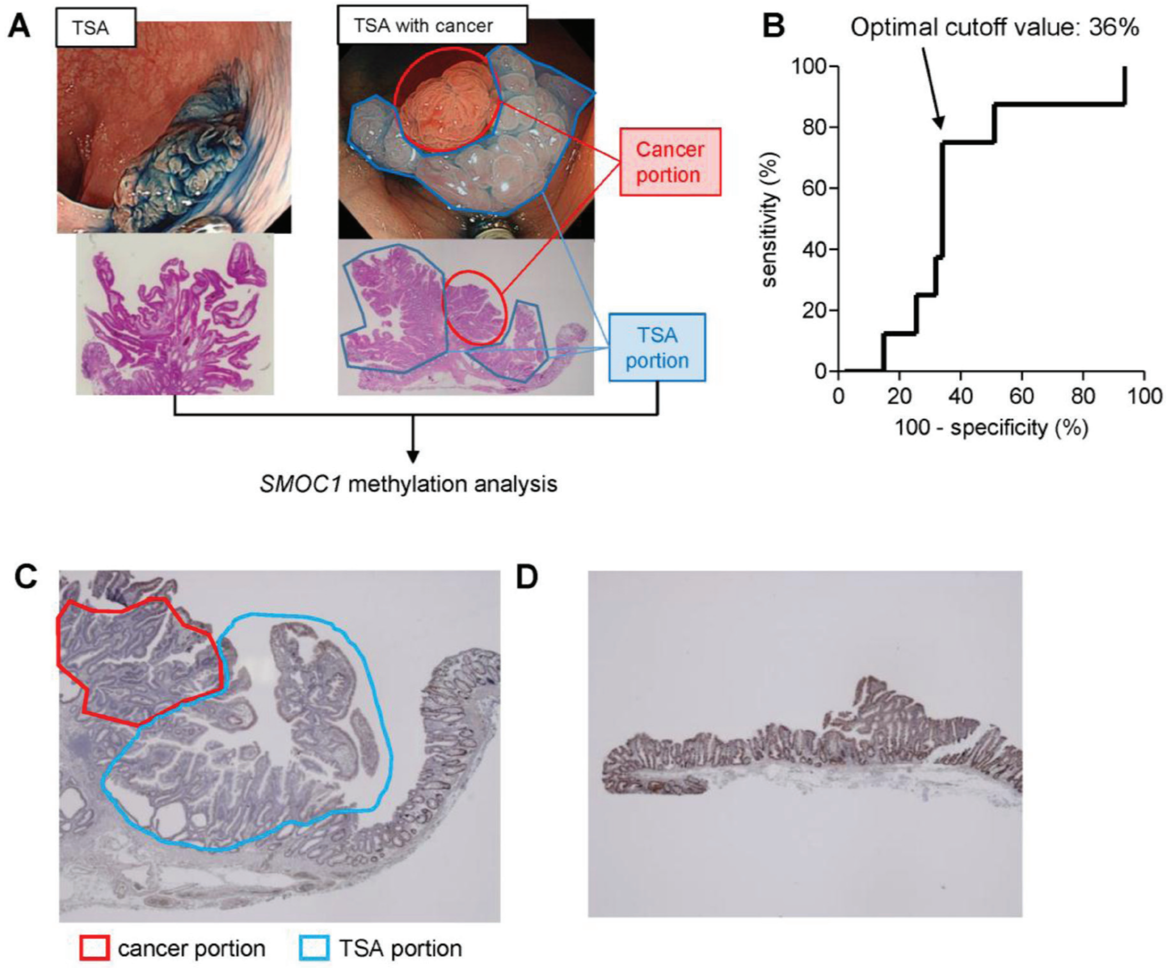

Supplementary Figure 5: *SMOC1* methylation is associated with malignant progression of TSAs. (**A**) Representative examples of a TSA (left) and a TSA with cancer (right). Endoscopic views are shown on the top, and histological views are below. (**B**) Receiver operating characteristic (ROC) curve analysis of the ability of *SMOC1* methylation to discriminate between TSAs and TSAs with cancer. The optimal cutoff point is also shown. (**C**) Immunohistochemical staining of SMOC1 in a representative specimen of TSA with cancer. Levels of *SMOC1* methylation in the TSA portion, the cancer portion and the non-tumorous portion were 38%, 55% and 7%, respectively. (**D**) Immunohistochemical staining of SMOC1 in a representative specimen of SSA/P with cytological dysplasia. The level of *SMOC1* methylation in this specimen was 7%.

Supplementary Table 1: Sequences of the primers used in this study. See Supplementtary_Table_1
